# Stereopsis and retinal microstructures following macular hole surgery

**DOI:** 10.1038/s41598-020-76648-4

**Published:** 2020-11-11

**Authors:** Fumiki Okamoto, Yuki Moriya, Yoshimi Sugiura, Tomoya Murakami, Shohei Morikawa, Sujin Hoshi, Takahiro Hiraoka, Tetsuro Oshika

**Affiliations:** Department of Ophthalmology, Faculty of Medicine, University of Tsukuba, 1-1-1 Tennoudai, Tsukuba, Ibaraki 305-8575 Japan

**Keywords:** Retinal diseases, Vision disorders

## Abstract

The aim of this prospective study was to evaluate the changes in stereopsis in patients who underwent vitrectomy for macular hole (MH) and assess the relationship between stereopsis and retinal microstructures. Fifty-two patients who underwent successful vitrectomy for unilateral MH and 20 control participants were recruited. We examined stereopsis using the Titmus Stereo Test (TST) and TNO stereotest (TNO), optical coherence tomography, and best-corrected visual acuity measurements, preoperatively, and at 3, 6, and 12 months postoperatively. As a result, preoperative and postoperative 3, 6, and 12-month values of stereopsis assessed by TST (log) were 2.7, 2.2, 2.2, and 2.2, respectively. TNO (log) were 2.8, 2.5, 2.4, and 2.4, respectively. Stereopsis in MH after surgery was significantly worse than that in normal participants (*p* < 0.001). Preoperative TST significantly correlated with MH size and defect length of external limiting membrane (ELM). Postoperative TST demonstrated significant correlation with the preoperative ELM defect length, and postoperative TNO was associated with the preoperative interdigitation zone defect length. Vitrectomy for MH significantly improved stereopsis, although not to normal levels. The ELM defect lengths, which approximately correspond to TST circles, are prognostic factors for postoperative stereopsis by TST. The interdigitation zone defect length, similar in size to the TNO index, is a prognostic factor for postoperative stereopsis by TNO.

## Introduction

In many patients, visual acuity improves after the successful surgical closure of the idiopathic macular hole (MH)^1,2^; however, some patients still experience disturbances in visual function after surgery. MH results in reduced visual acuity, metamorphopsia^3–9^, and aniseikonia^10,11^.

Stereopsis is the ability to perceive the depth of the field based on the disparity in the images formed in each eye—it is the most advanced visual function. Several studies have reported the relationship between stereopsis and retinal disorders, such as MH^12–14^, epiretinal membrane (ERM)^14,15^, and retinal detachment (RD)^16^, and found that vitrectomy improved stereopsis in patients with MH^12,13^.

With the introduction of optical coherence tomography (OCT), clinicians can evaluate the microstructural changes in the macular region qualitatively and quantitatively^17,18^. Several studies have examined the relationship between OCT findings and visual acuity^19–25^, metamorphopsia^4–9^, and aniseikonia^10,11^, in patients with MH. However, to our best knowledge, no study has prospectively investigated the temporal changes in stereopsis or the relationship between stereopsis and OCT findings in patients with MH.

The purpose of the present study was to evaluate stereopsis in patients who underwent vitrectomy for MH and to investigate the relationship between stereopsis and retinal microstructures.

## Methods

We analysed 52 patients (18 men and 34 women) who underwent successful vitrectomy surgery for treating unilateral MH, and 20 age-matched normal participants (8 men and 12 women) at the Tsukuba University Hospital. The average age of the patients and controls was 66.7 ± 6.7 and 65.5 ± 6.0 years (mean ± standard deviation), respectively. We conducted this prospective, comparative study in accordance with the Declaration of Helsinki and received approval from the institutional review committees of the University of Tsukuba Hospital. All the patients provided informed consent after the nature of the study was explained to them before inclusion in the study. The exclusion criteria included eyes with MH secondary to uveitis or proliferative diabetic retinopathy, traumatic MH, ophthalmic diseases except mild cataract and refractive errors, and any systemic disease that influenced ocular motility.

The examinations included measurements of stereopsis, best-corrected visual acuity (BCVA), and retinal microstructure before surgery and at 3, 6, and 12 months after surgery. Stereopsis was measured using the Titmus Stereo Test (TST) and TNO stereotest (TNO). These tests were performed at a distance of 40 cm with appropriate spectacle correction. The results of stereopsis tests were expressed as ‘seconds of arc’. We converted these values to logarithms for statistical evaluation.

**Figure 1:**
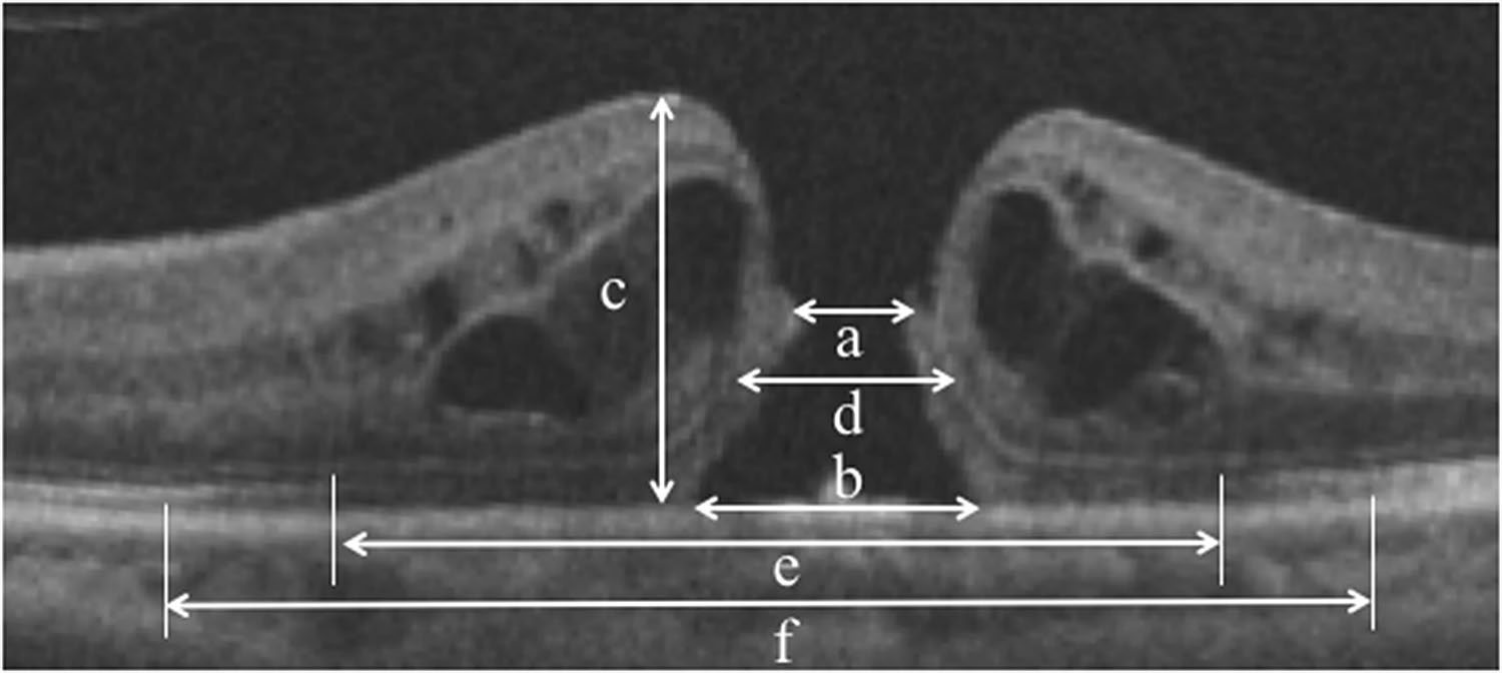
Based on preoperative optical coherence tomography images in patients with macular hole (MH), we measured the minimum MH diameter (**a**), base MH diameter (**b**), macular thickness (**c**), defect lengths of the external limiting membrane (**d**), ellipsoid zone (**e**), and interdigitation zone (**f**). We quantified the parameters with an image processing software (ImageJ bundled with Java 1.8.0_172, developed by Wayne Rasband, National Institutes of Health, Bethesda, MD; available from https://rsbweb.nih.gov/ij/index.html).

The retinal microstructure was measured with spectral-domain OCT (Cirrus high-definition OCT; Carl Zeiss, Dublin, CA, USA). We used 5-line raster scans for each eye using an analytical software package (Cirrus analysis software, version 3.0; Carl Zeiss) with a signal strength of more than 7/10. We quantified the following parameters before surgery based on OCT images: minimum diameters of MH, base diameters of MH, macular thickness, and external limiting membrane (ELM), ellipsoid zone (EZ), and interdigitation zone (IZ) defect lengths (Fig. 1)^8^. Based on the images of the 5-line raster scans, we quantified the parameters with an image processing software (ImageJ bundled with Java 1.8.0_172, developed by Wayne Rasband, National Institutes of Health, Bethesda, MD; available from https://rsbweb.nih.gov/ij/index.html)^8^. The defect length of each line was determined by agreement between two blinded, well-trained observers (Y.S. and Y.M.), and the mean value of the length of each line was used for further analysis.

All surgeries were performed by two surgeons (F.O. and Y.O.) under sub-Tenon’s local anaesthesia. Clinically significant cataracts were simultaneously operated. We induced posterior vitreous detachment and performed core vitrectomy. Subsequently, we injected 0.2 mL of 0.025% brilliant blue G solution over the macula for 10–20 s and thereafter, washed it with an irrigation solution. Internal limiting membrane (ILM) peeling and fluid/gas exchange were performed for all cases. The patients were instructed to be in a face-down position for 1–3 days postoperatively.

The mean scores were compared, and standard deviations were calculated for each parameter of visual function and OCT measurement. BCVA was measured with the Landolt Chart and expressed as a logarithm of the minimum angle of resolution. The Mann–Whitney *U* test was used to compare stereopsis between patients with MH and normal participants. The Wilcoxon signed rank test was used to compare differences in stereopsis before and after surgery in patients with MH. Repeated-measures analysis of variance was used to clarify the changes in visual acuity and stereopsis. When a significant difference was detected, we conducted the Dunnett post hoc test for multiple comparisons to reveal the time point that demonstrated a significant difference from the baseline value. The associations between stereopsis and OCT parameters and visual acuity were examined by the Spearman rank correlation test. All tests for evaluating associations between parameters were considered statistically significant if *P* was < 0.05. All analyses were performed using StatView (version 5.0, SAS Inc., Cary, NC, USA).

## Results

Table 1 reveals the visual functions and OCT parameters of patients with MH before surgery. Preoperatively, 40 eyes were phakic and 12 eyes were pseudophakic with posterior chamber intraocular lenses. All phakic eyes were performed with cataract surgery combined with vitrectomy.

**Table 1:** Clinical Features of visual functions and OCT parameters in patients with macular hole (MH) before surgery.

Age (years)	66.7 ± 6.7
Sex (men / women)	18 / 34
Preoperative best-corrected visual acuity (logMAR)	0.72 ± 0.34
Stage of MH (2 / 3 / 4)	(15 / 25 / 12)
Titmus Stereo Test (TST) (log)	2.7 ± 0.5
TNO stereotest (log)	2.8 ± 0.6
Minimum diameters of MH (µm)	394 ± 174
Base diameter of MH (µm)	796 ± 266
Macular thickness (µm)	445 ± 123
Defect length of external limiting membrane (ELM) (µm)	755 ± 322
Defect length of ellipsoid zone (EZ) (µm)	1320 ± 504
Defect length of interdigitation zone (IZ) (µm)	2126 ± 421

Values are presented as the mean ± standard deviation.

LogMAR = logarithm of the minimum angle of resolution.

OCT: optical coherence tomography.

### Pre- and postoperative stereopsis in patients with MH and normal participants

The preoperative and postoperative 3, 6, and 12-month values of stereopsis assessed by TST (log) were 2.7 ± 0.5, 2.2 ± 0.3, 2.2 ± 0.3, and 2.2 ± 0.4, respectively. The preoperative and postoperative 3, 6, and 12-month values of stereopsis assessed by TNO (log) were 2.8 ± 0.6, 2.5 ± 0.5, 2.4 ± 0.5, and 2.4 ± 0.4, respectively. TST and TNO values (log) in normal participants were 1.6 ± 0.1 and 1.9 ± 0.2, respectively. Stereopsis values in patients with MH before surgery and 12 months after surgery were significantly worse than those of normal participants (*p* < 0.001).

### Temporal changes in visual function in patients with MH

**Figure 2:**
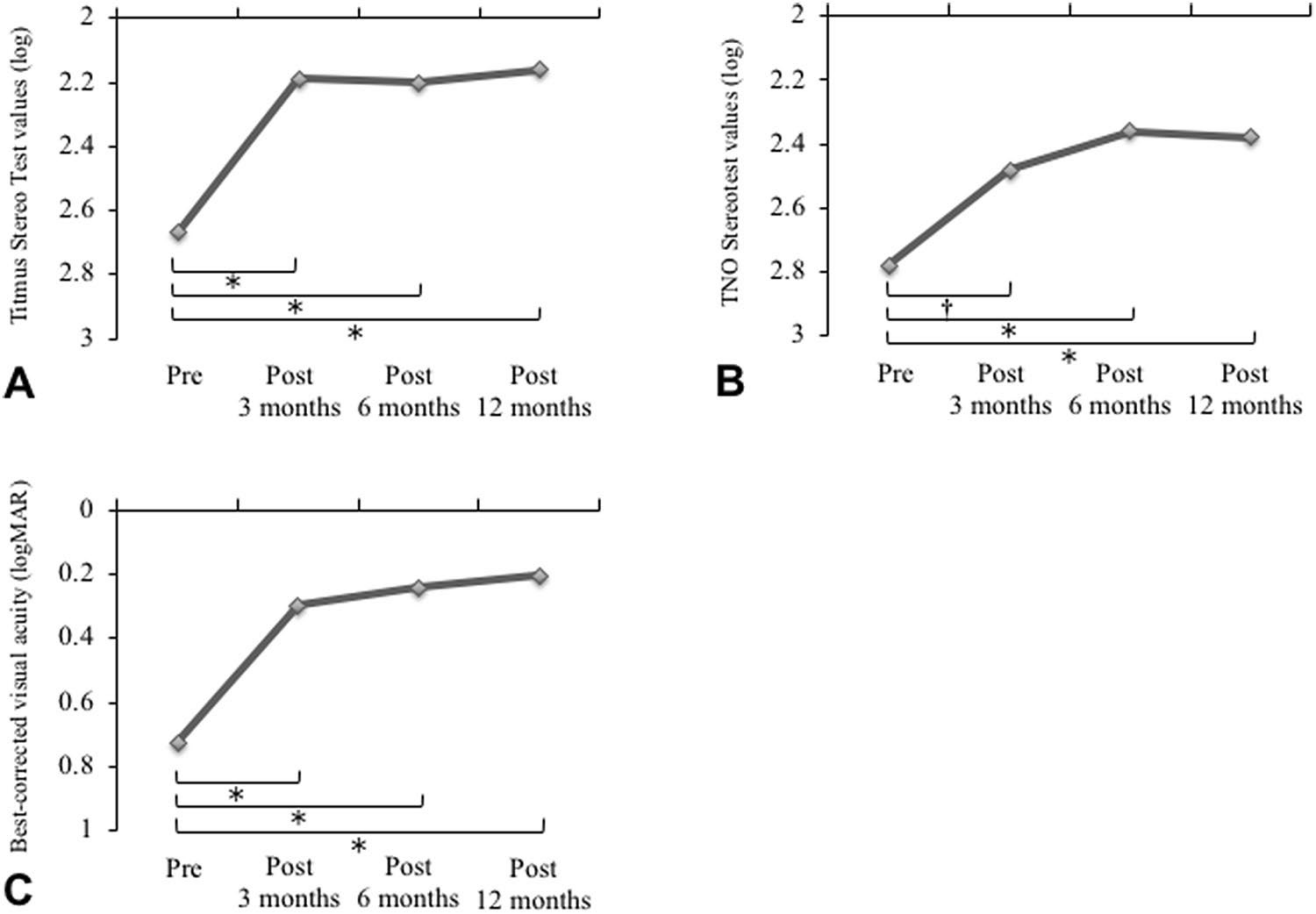
Temporal changes in visual function in patients with macular hole before and after surgery. (**A**) Titmus Stereo Test values. (**B**) TNO stereotest values. (**C**) Best-corrected visual acuity. Visual functions significantly improved after surgery. *P < 0.0001; ^†^*P* < 0.001.

Vitrectomy improved visual acuity and stereopsis. The preoperative TST values were significantly higher than those observed at 3, 6, and 12 months postoperatively. No significant differences were observed between the TST values at 3 and 6 months or at 6 and 12 months postoperatively (Fig. 2A). Similar results were found for TNO (Fig. 2B). The preoperative and postoperative 3, 6, and 12-month values of BCVA were 0.72 ± 0.34, 0.30 ± 0.27, 0.24 ± 0.25, and 0.20 ± 0.19, respectively. Preoperative BCVA was significantly higher than that measured 3, 6, and 12 months postoperatively (Fig. 2C). Postoperative TST at 12 months was significantly correlated with preoperative TST (r = 0.53, *P* < 0.001), while postoperative TNO at 12 months did not show correlation with preoperative TNO (r = 0.22, *P* = 0.12).

### Relationship between stereopsis and visual acuity in patients with MH

Preoperative stereopsis assessed by TST showed a significant correlation with preoperative BCVA (r = 0.32, *P* < 0.05), but stereopsis assessed by TNO was not associated with preoperative BCVA (r = 0.21, *P* = 0.13). At 3 months postoperatively, stereopsis was not associated with BCVA (TST: r = 0.17, *P* = 0.23; TNO: r = 0.15, *P* = 0.28). At 6 months postoperatively, TST and TNO was not also correlated with BCVA (TST: r = 0.22, *P* = 0.11; TNO: r = 0.21, *P* = 0.14). Stereopsis was significantly correlated with BCVA (TST: r = 0.53, *P* < 0.0001; TNO: r = 0.34, *P* < 0.05) at 12 months postoperatively. Postoperative TST values significantly correlated with preoperative BCVA (r = 0.30, *P* < 0.05), while postoperative TNO values showed no association with preoperative BCVA (r = 0.19, *P* = 0.18).

### Relationship between stereopsis and OCT parameters before surgery

The preoperative TST values showed significant correlation with the minimum and base diameters of MH (*P* < 0.01 and *P* < 0.01, respectively) and with the ELM defect length (*P* < 0.01). TNO values were significantly associated with the base diameter of MH (*P* < 0.05; Table 2).

**Table 2:** Correlation between preoperative stereopsis and OCT parameters in patients with macular hole (MH).

Preoperative parameters	Preoperative TST		Preoperative TNO	
	r	p	r	p
Minimum diameter of MH	0.41	0.006*	0.27	0.07
Base diameter of MH	0.40	0.005*	0.30	0.04*
Macular thickness	0.20	0.20	0.07	0.74
Defect length of ELM	0.39	0.007*	0.24	0.12
Defect length of EZ	0.16	0.33	0.05	0.89
Defect length of IZ	0.22	0.16	0.21	0.18

*Significant correlation between parameters (Spearman rank correlation test).

TST = Titmus Stereo Test, TNO = TNO stereotest, ELM = external limiting membrane, EZ = ellipsoid zone, IZ = interdigitation zone.

OCT: optical coherence tomography, MH: macular hole.

### Preoperative OCT parameters affecting postoperative stereopsis

**Figure 3:**
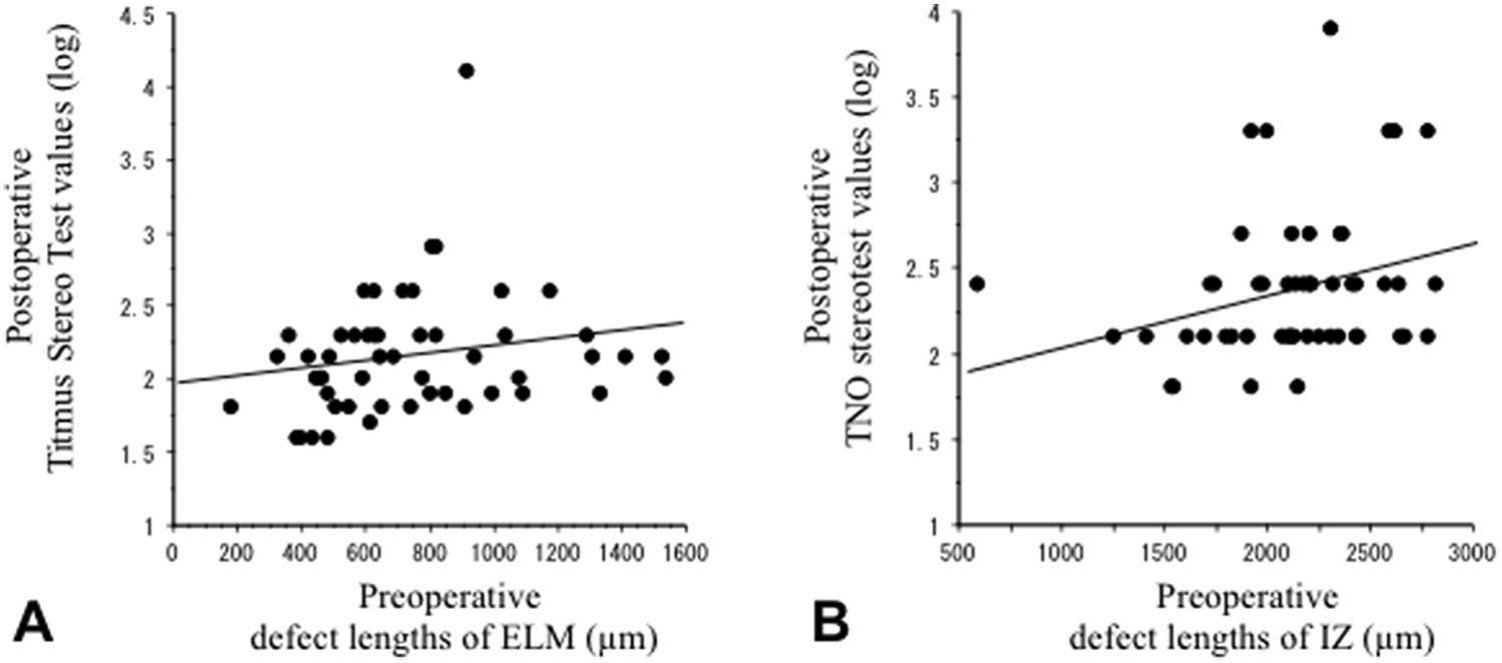
Correlation between postoperative stereopsis and preoperative optical coherence tomography parameters. (**A**) Postoperative Titmus Stereo Test values versus preoperative defect lengths of the external limiting membrane in patients with macular hole. (**B**) Postoperative TNO stereotest values versus preoperative defect lengths of the interdigitation zone. ELM, external limiting membrane; IZ, interdigitation zone.

Table 3 shows the relationship between the postoperative 12-month values of stereopsis and preoperative OCT parameters in patients with MH. The postoperative 12-month TST values significantly correlated with preoperative ELM defect length (*P* < 0.05, Fig. 3A). Postoperative TNO values showed a significant correlation with preoperative IZ defect length (*P* < 0.05, Fig. 3B).

**Table 3:** Correlation between 12-month postoperative stereopsis and preoperative OCT parameters in patients with macular hole (MH).

Preoperative parameters	Postoperative TST		Postoperative TNO	
	r	p	r	p
Minimum diameter of MH	0.19	0.19	0.07	0.85
Base diameter of MH	0.25	0.09	0.08	0.81
Macular thickness	0.01	0.99	0.06	0.98
Defect length of ELM	0.29	0.04*	0.17	0.35
Defect length of EZ	0.21	0.16	0.17	0.35
Defect length of IZ	0.23	0.12	0.32	0.04*

*Significant correlation between parameters (Spearman rank correlation test).

TST = itmus Stereo Test, TNO = NO stereotest, ELM = external limiting membrane, EZ  = ellipsoid zone, IZ = nterdigitation zone.

OCT: optical coherence tomography.

## Discussion

The postoperative visual acuity in patients with MH tended to improve gradually over 1 year, while stereopsis improved immediately within 3 months after surgery, after which it remained unchanged. Stereopsis is a more advanced visual function than visual acuity, and there may be a limit to the extent of surgical improvement of stereopsis. It has already been reported that stereopsis improves after surgery for treating ERM and MH;^12–15^; however, these studies did not report detailed temporal changes in stereopsis. This is the first study to report temporal changes in stereopsis in patients with MH.

Stereopsis was compromised in patients with MH and was worse in patients with MH than that in normal controls in this study. Previous studies have investigated disturbances in stereopsis in retinal disorders, including unilateral ERM and RD, and compared them with stereopsis assessed in normal controls^15,16^. Stereopsis was affected in patients with MH similar to that in patients with other unilateral retinal diseases^14^. Moreover, stereopsis was impaired in patients with MH after surgery, compared with that in controls. Stereopsis improved following surgical treatment for ERM and RD^15,16^, although not to the values found in normal individuals. Despite treatment, patients with MH presented with poor stereopsis similar to that observed in patients with other retinal diseases.

Stereopsis assessed by TST was associated with visual acuity in patients with MH. The relationship between visual acuity and stereopsis has been investigated in normal participants^26,27^ and in patients who underwent surgery for MH^12,13^ and RD^28^. Lam et al.^29^ reported a difference in visual acuity between the eyes as a cause of disturbance of stereopsis, while Burian^30^ reported that stereopsis deteriorated with worsening visual acuity in one eye. Judging from these findings, stereopsis, which is binocular vision, is impaired even if visual acuity in one eye is impaired. We included MH patients with one eye and normal vision in the other eye in this study. It is consistent with the results of these articles that worsening visual acuity in one eye impairs stereopsis.

In contrast, preoperative stereopsis assessed by TNO was not significantly associated with visual acuity in patients with MH. This difference may be attributed to the different index sizes used in the two stereotests. The stimulus used for assessing fine stereopsis in TNO was much larger than that used in the TST circles. The TST circles subtend a visual angle of 0.7°, while those in TNO subtend an angle of 8.5°. The diameters of the fovea and foveola were 1500 and 350 μm, respectively, with visual angles of approximately 5° and 1.2°, respectively. Therefore, TST circles are more relevant to visual acuity than TNO. Generally, MH patients do not complain of stereoscopic vision. This may be because the central stereopsis deficit indicated by TST is supplemented by paracentral stereopsis indicated by TNO in the visual field pathway in the brain.

Preoperative stereopsis had a significant correlation with various OCT parameters, including the minimum diameter of MH, base diameter of MH, and ELM defect length. Several studies have investigated the relationship between visual functions and OCT findings in patients with MH. Visual acuity was significantly associated with the minimum and base diameters of MH^19^, and the EZ^20–23^, and IZ defect lengths^24,25^. Moreover, metamorphopsia was associated with the minimum and base diameters of MH^6,7^, asymmetric elongation of foveal tissue^9^, and intraretinal cysts within the fluid cuff^8^. Stereopsis was related to the size of the MH, outer retinal layers, visual acuity, and metamorphopsia.

**Figure 4:**
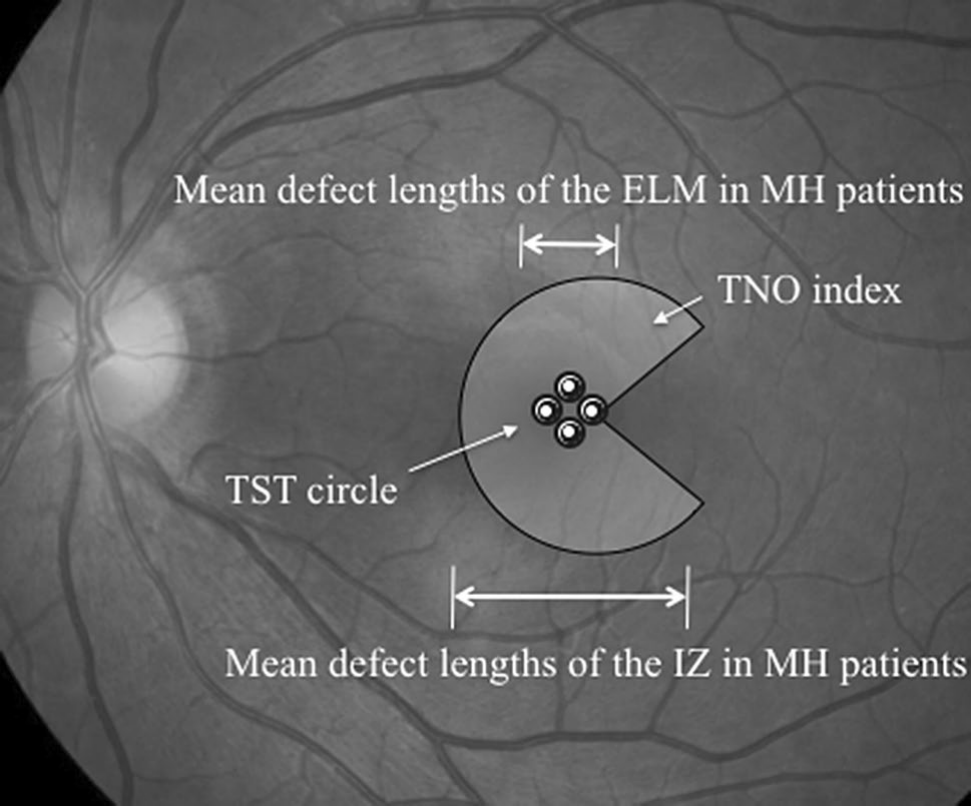
Approximate projection of Titmus Stereo Test circles and TNO stereotest index of the macula in relation to the mean defect lengths of the external limiting membrane and interdigitation zone in patients with macular hole. ELM, external limiting membrane; IZ, interdigitation zone; MH, macular hole; TST, Titmus Stereo Test; TNO, TNO stereotest.

The prognostic factors for the assessment of postoperative stereopsis in patients with MH were ELM defect length by TST and IZ defect length by TNO. The difference in the prognostic factors between TST and TNO is probably due to the different index sizes used in the two tests as mentioned previously. Generally, the defect length of IZ is longer than that of ELM because of the shape of the MH. In this study, the mean defect length of ELM was 755 µm and that of IZ was 2126 µm. Interestingly, when the indices and results of defect lengths of the two tests are superimposed on the fundus photograph, the indices and defect lengths of the two tests coincide (Fig. 4). Therefore, the extent of impairment of retinal structures was reflected in the results of stereopsis.

Our study has several limitations, especially the small sample size and short follow-up duration. Visual acuity and stereopsis in patients with MH may improve over the course of a longer follow-up period. Although factors such as eye dominance^31,32^, pupil size^33,34^, and accommodation^26,35^, are known to affect stereopsis, we did not evaluate these factors in this study. In addition, our study population was relatively small MH patients (Minimum diameters of MH was 394 µm in average). The present results may not be totally suitable for larger macular holes. Future studies that include a larger sample size, longer follow-up duration, and evaluate other factors will further improve our understanding of stereopsis and other visual functions in patients with MH.
